# The host genetic background defines diverse immune-reactivity and susceptibility to chronic *Pseudomonas aeruginosa* respiratory infection

**DOI:** 10.1038/srep36924

**Published:** 2016-11-16

**Authors:** Lorenza Spagnuolo, Maura De Simone, Nicola Ivan Lorè, Ida De Fino, Veronica Basso, Anna Mondino, Cristina Cigana, Alessandra Bragonzi

**Affiliations:** 1 Infection and Cystic Fibrosis Unit, IRCCS San Raffaele Scientific Institute, Milano, Italy; 2 Lymphocyte activation Unit, IRCCS San Raffaele Scientific Institute, Milano, Italy

## Abstract

Patients with *P. aeruginosa* airways infection show markedly variable clinical phenotypes likely influenced by genetic backgrounds. Here, we investigated the cellular events involved in resistance and susceptibility to *P. aeruginosa* chronic infection using genetically distinct inbred mouse strains. As for patients, different murine genotypes revealed variable susceptibility to infection. When directly compared, resistant C3H/HeOuJ and susceptible A/J strains revealed distinct immune responsiveness to the pathogen. In C3H/HeOuJ resistant mice, IL17-producing cells rapidly and transiently infiltrated the infected lung, and this was paralleled by the acute accumulation of alveolar macrophages, bacterial clearance and resolution of infection. In contrast, A/J susceptible mice revealed a more delayed and prolonged lung infiltration by IL17^+^ and IFNγ^+^ cells, persistence of innate inflammatory cells and establishment of chronic infection. We conclude that the host genetic background confers diverse immunoreactivity to *P. aeruginosa* and IL17-producing cells might contribute to the progress of chronic lung infection.

Patients with cystic fibrosis (CF) and chronic obstructive pulmonary disease (COPD) experience chronic pulmonary colonization with *Pseudomonas aeruginosa.* This bacterial infection often develops with acute exacerbations that have a major impact on the patients’ quality of life and mortality. However, the onset and the clinical outcome of *P. aeruginosa* infections are extremely variable among patients at risk. In COPD, only a subset of adult patients (4–15%) develops *P. aeruginosa* chronic infection^1^. In the CF genetic disease, the great majority of patients (80%) carries *P. aeruginosa* infection but the progression and severity of pulmonary disease is not strictly correlated with the *CF transmembrane conductance regulator (CFTR*) gene mutations^2^. Thus, considerable inter-individual variability in *P. aeruginosa* susceptibility, which is difficult to explain in the clinical context, creates a great challenge for the clinicians in managing treatment for patients.

It is recognized that the genotype is an important determinant of host susceptibility to major human diseases, including infections^3^,^4^. How the host genetic background mediates disease outcome and pathogenesis of chronic lung infection by shaping the host response to *P. aeruginosa* remains to be established. Phenotype/genotype correlations are difficult to approach in humans, while phylogenetically different strains of mice have been successfully used to clarify aspects of different susceptibility to *P. aeruginosa* infection^5^,^6^,^7^,^8^,^9^,^10^. In mouse strains presenting deviant clinical phenotypes, a faster and higher cell-mediated innate immune response sets the conditions for resistance during early *P. aeruginosa* infection, as opposed to susceptible phenotype^9^. Whether, how and at which extent cell mediated immunity, in particular T lymphocytes, also plays a role in chronic infection remains to be elucidated.

Until recently, resistance/susceptibility to *P. aeruginosa* infection were uniquely linked to the capacity of the host to mount a type 1 or type 2 immune response, respectively. In CF patients, a type 1 immunity was associated with improved pulmonary functions while the suboptimal representation of IFNγ producing Th cells was linked to more frequent *P. aeruginosa* infections^11^. Moreover, a Th2 response was frequently found in *P. aeruginosa* chronically infected CF individuals and associated with poor clinical status^12^,^13^. Similarly, *P. aeruginosa*-resistant murine strains were Th1 responders (C3H/HeN), while the susceptible ones were Th2 responders (BALB/c)^14^,^15^.

More recently, Th17 T cells have been described in CF patients with chronic *P. aeruginosa* infection, and Th17 cytokines (IL17A, IL17F, IL23) were markedly elevated in bronchoalveolar lavage fluid (BALF) or sputum of CF patients undergoing pulmonary exacerbation^16^. Similar trends were also observed in sputum of CF patients clinically stable and infected by *P. aeruginosa*^17^. In murine models, IL17/IL23-induced signaling was involved in the generation of the inflammatory response^18^ and IL17 was demonstrated to play a protective role in acute and chronic pulmonary *P. aeruginosa* infection^17^,^19^,^20^,^21^.

Thus, together available evidences indicate that both innate and adaptive immune responses play a role over the course of *P. aeruginosa* infection, which might vary according to the host genetic background. Detailed analysis of host immune responses to *P. aeruginosa* during long-term chronic colonization have to date being hampered by the absence of an animal model reliably mimicking the course of human infection. We recently refined and characterized such model allowing for *P. aeruginosa* long-term chronic colonization and dissection of the cell-mediated immunity^17^,^21^. Here we exploited this model to screen phylogenetically different mouse strains and test susceptibility/resistance to the establishment of *P. aeruginosa* chronic infection, and also longitudinally characterize innate and adaptive immunity to the pathogen. We report that the host genetic background confers different immunoreactivity to *P. aeruginosa* chronic infection, and that delayed IL17-producing cells responses might contribute to the predisposition to chronic infection.

## Results

### Clinical phenotypes in *P. aeruginosa*-resistant and susceptible mice during the chronic lung infection

**Figure 1:**
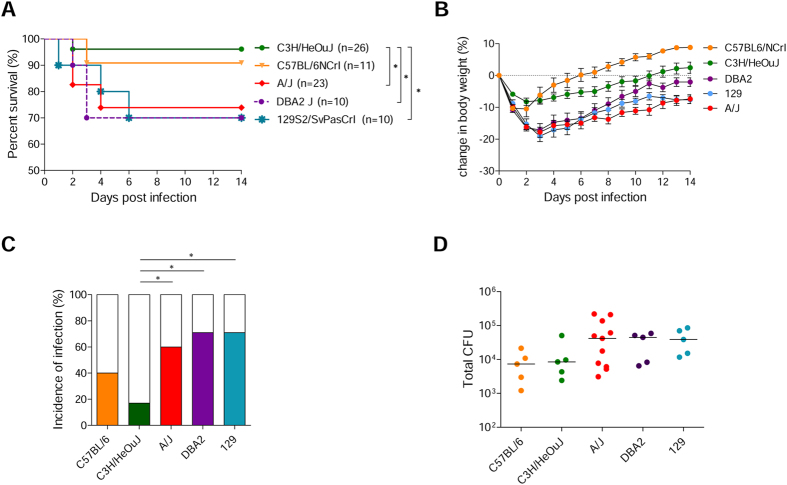
Survival, body weight, percentage of chronicity and pulmonary bacterial burden after *P. aeruginosa* infection in inbred mouse strains. DBA/2 J (n = 10), 129S2/SvPasCRL (n = 12), A/J (n = 24), C3H/HeOuJ (n = 26) and C57BL/6NCrl (n = 11) mice were inoculated with 2 × 10^6^ CFU of *P. aeruginosa* mucoid clinical isolate AA43 embedded in agar beads by intratracheal injection, and monitored for survival (**A**) and body weight change (**B**) for a period of 14 days after challenge. In addition, the clearance (white) and rate of chronic colonization (colored) (**C**) and the bacterial burden in the lungs (**D**) after 14 days from *P. aeruginosa* challenge were determined in surviving mice. (**B**) The error bars represent the standard error of the mean (SEM). (**D**) Dots represent lung CFU in individual mice and horizontal lines represent median values reported in log scale. The data are pooled from two to three independent experiments. Statistical significance by Mantel-Cox test (**A**), Fisher’s test followed by Bonferroni correction for multiple comparisons (**C**), and Mann-Whitney U test and nonparametric Kruskal-Wallis test followed by post-hoc Dunn test to correct for multiple comparisons (**D**) is indicated: *p < 0.05. Two-way ANOVA with Bonferroni’s post-hoc test (**B**) is reported in Table S1.

DBA/2 J, 129S2/SvPasCrl, A/J, C3H/HeOuJ and C57BL/6NCrl were chosen based on their different susceptibility to acute and lethal *P. aeruginosa* infection^9^ and were challenged via intra-tracheal injection with 2 × 10^6^ CFUs of the *P. aeruginosa* AA43 adapted clinical isolate embedded in agar beads. In previous studies, we found this strategy to result in the establishment of chronic infection, highly reminiscent of the human pathology^21^,^22^. DBA/2 J, 129S2/SvPasCrl and A/J mice revealed lower survival rates and more marked weight losses in the early stage of the *P. aeruginosa* infection when compared to C57BL/6NCrl and C3H/HeOuJ mice, that instead revealed better survival rates and lower morbidity (Fig. 1A,B and Supplementary Tables 2 and 3).

Next, we evaluated the predisposition to the establishment of long-term chronic infection. At 14 days, susceptible A/J, DBA/2 J and 129S2/SvPasCrl mice showed significantly higher incidence of chronic infection when compared to the resistant C3H/HeOuJ strain, while the C57BL/6NCrl strain showed an intermediate phenotype (Fig. 1C and Supplementary Table 3). In addition, although not statistically different, in average pulmonary bacterial loads were 1 Log higher in susceptible A/J, DBA/2 J, and 129S2/SvPasCrl than in C3H/HeOuJ and C57BL/6NCrl (Fig. 1D and Supplementary Table 3).

**Figure 2:**
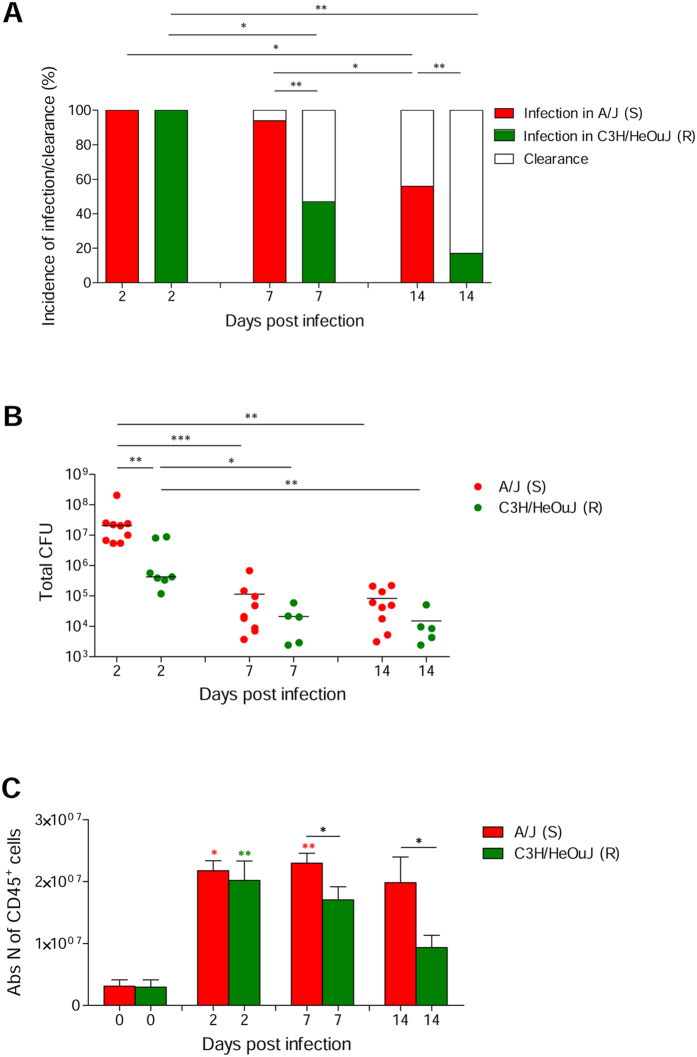
Percentage of infected mice, pulmonary bacterial burden and level of leukocyte recruitment (CD45^+^ cells) during chronic *P. aeruginosa* lung infection in resistant and susceptible mice. Susceptible A/J and resistant C3H/HeOuJ mice were inoculated with 2 × 10^6^ CFU of *P. aeruginosa* mucoid clinical isolate AA43 embedded in agar beads by intratracheal injection and sacrificed at day 2, 7 and 14 post challenge. At each time point, the clearance (white) or rate of chronic colonization (colored) (**A**) and the bacterial burden in the lungs (**B**) were evaluated. Dots represent CFU per lung in individual mice and horizontal lines represent median values reported in log scale (**B**). Leukocytes (CD45^+^ cells) were measured in lung cell suspension by flow cytometric analysis (**C**). The error bars represent the SEM (**C**). The data are pooled from two to three independent experiments. Statistical significance by Fisher’s test followed by Bonferroni correction for multiple comparisons (**A**), and Mann-Whitney U test and nonparametric Kruskal-Wallis test followed by post-hoc Dunn test to correct for multiple comparisons (**B**,**C**) is indicated: *p < 0.05, **p < 0.01, ***p < 0.001. Colored stars indicate the statistical significance of the difference between each mouse strain at the specific time point compared with its own naïve counterpart.

**Figure 3:**
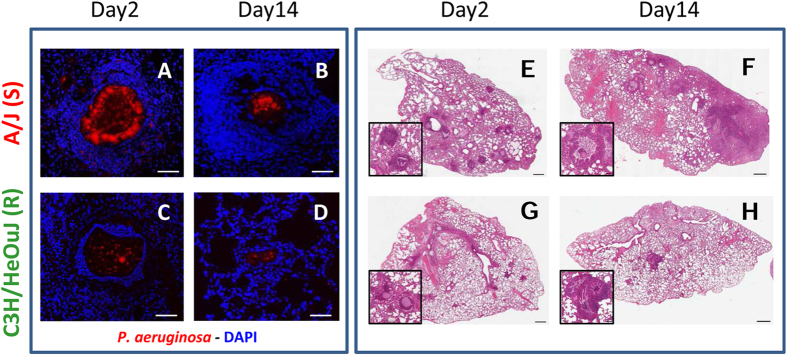
Lung histopathology and *P. aeruginosa* immunofluorescence staining during chronic infection in resistant and susceptible mice. Susceptible A/J and resistant C3H/HeOuJ mice were inoculated with 2 × 10^6^ CFU of *P. aeruginosa* mucoid clinical isolate AA43 embedded in agar beads by intratracheal injection and sacrificed at day 2, 7 and 14 post challenge. The figure exemplifies the lungs of A/J and C3H/HeOuJ mice stained in immunofluorescence with specific antibody against *P. aeruginosa* (red) (**A–D**) and with H&E (**E–H**). Counterstaining for immunofluorescence was performed with 49,6-Diamidino-2-phenylindole dihydrochloride (DAPI) (blue). Bars: 100 μm for immunofluorescence images and 500 μm for H&E images.

The most susceptible and resistant mice (i.e. A/J and C3H/HeOuJ, respectively) were further characterized in time course analyses. At day 2, both A/J and C3H/HeOuJ mice held *P. aeruginosa* in the lungs (Fig. 2A). Nevertheless, by this time, resistant C3H/HeOuJ mice displayed a lower bacterial burden (4.2 × 10^5^ CFUs) compared to susceptible A/J mice (2.1 × 10^7^ CFUs) (Fig. 2B). Although by day 7 and 14, the number of infected mice and the pulmonary bacterial load decreased in both strains, the frequency of chronically infected mice was significantly higher in A/J mice than in C3H/HeOuJ (day 7: 94% versus 47%; day 14: 56% versus 17%) (Fig. 2A). Of note, although CD45^+^ inflammatory cells equally infiltrated lungs of infected mice by day 2, they persisted to higher numbers in chronically infected A/J susceptible mice when compared to chronically infected C3H/HeOuJ resistant mice (Fig. 2C). Immunofluorescence staining and histopathological analysis showed the persistence of higher number of *P. aeruginosa* cells in A/J mice when compared to C3H/HeOuJ (Fig. 3A–D), and the presence of persistent and severe inflammatory lesions (Fig. 3E,F) in the A/J mice and rather signs of progressive resolution of inflammation in C3H/HeOuJ (Fig. 3G,H).

When measuring pulmonary cytokines/chemokines, we found that several of them were expressed at higher levels in the lungs of A/J susceptible mice compared to resistant C3H/HeOuJ early after infection (day 2) (Table 1), likely reflecting higher bacterial loads (Fig. 2B). By day 7, cytokines/chemokines expression decreased in both strains, although at slower rate in susceptible mice compared to resistant, to reach similar levels at day 14 (Table 1).

Thus, as reported in humans, also inbred mouse strains reveal different susceptibility to infection, with C3H/HeOuJ and A/J being the most resistant and susceptible ones, respectively, and diverse pulmonary pathology.

### Pulmonary innate immune response in *P. aeruginosa-*susceptible and resistant mice

**Figure 4:**
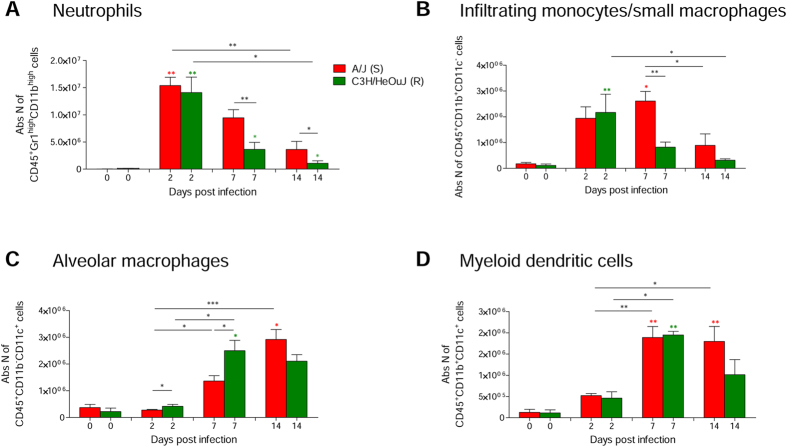
Levels of neutrophils, infiltrating monocytes/small macrophages, alveolar macrophages and myeloid dendritic cells during chronic *P. aeruginosa* infection in resistant and susceptible mice. Susceptible A/J and resistant C3H/HeOuJ mice were inoculated with 2 × 10^6^ CFU of *P. aeruginosa* mucoid clinical isolate AA43 embedded in agar beads by intratracheal injection and sacrificed at day 2, 7 and 14 post challenge. The absolute numbers of neutrophils (CD45^+^Gr1^high^CD11b^high^) (**A**), infiltrating monocytes/small macrophages (CD45^+^CD11b^+^CD11c^−^) (**B**), alveolar macrophages (CD45^+^CD11b^−^ CD11c^+^) (**C**) and myeloid dendritic cells (mDCs) (CD45^+^CD11b^+^CD11c^+^) (**D**) were measured by flow cytometric analysis in lung cell suspension of naïve mice and after 2, 7 and 14 days post challenge and represented as bars. Bars represent mean values and the error bars the SEM. The data are pooled from two independent experiments. Statistical significance by Mann-Whitney U test and nonparametric Kruskal-Wallis test followed by post-hoc Dunn test to correct for multiple comparisons is indicated: *p < 0.05, **p < 0.01, ***p < 0.001. Colored stars indicate the statistical significance of the difference between each mouse strain at the specific time point compared with its own naïve counterpart.

To understand whether susceptibility/resistance to infection could be linked to host immune reactivity, we performed FACS-assisted immunophenotyping of lung CD45^+^ infiltrates during *P. aeruginosa* infection. By day 2 neutrophils, monocytes, and macrophages were all represented within infected lungs. Neutrophils (CD45^+^Gr1^high^CD11b^high^) and to a lower extent monocytes/small macrophages (CD45^+^CD11b^+^CD11c^−^) numbers decreased thereafter, and yet persisted to higher numbers in A/J susceptible mice (Fig. 4A,B, Supplementary Fig. 1A,B). Of note, C3H/HeOuJ and A/J-derived bone marrow neutrophils and peritoneal macrophages displayed similar *in vitro* phagocytic activity (Fig. S2), indicating that different bacterial burden are unlike to be attributable to defective phagocytic capacity of susceptible mice. Alveolar macrophages (CD45^+^CD11b^−^CD11c^+^; Fig. 4C, Supplementary Fig. 1C) and myeloid dendritic cells (CD45^+^CD11b^+^CD11c^+^; Fig. 4D, Supplementary Fig. 1D) accumulated in the infected lung by day 7. Of note, at 2 and 7 days lungs of resistant C3H/HeOuJ mice contained a significantly higher fraction of alveolar macrophages when compared to those derived from susceptible A/J mice, supporting their direct involvement in host defense and acute resolution of the airways inflammation (Fig. 4C, Supplementary Fig. 1C).

Together these data indicate that shortly after infection the lungs of both susceptible and resistant strains are infiltrated by component of the innate immune response, evoking an inflammatory response. Data indicate that alveolar macrophages best accumulate in the C3H/HeOuJ resistant strains at early time points, and this is associated to a better bacterial clearance. This event might be defective in A/J mice, where infection persists contributing to establishment of chronicity.

### Lymphocytes-mediated pulmonary immune response in *P. aeruginosa-*susceptible and resistant mice

**Figure 5:**
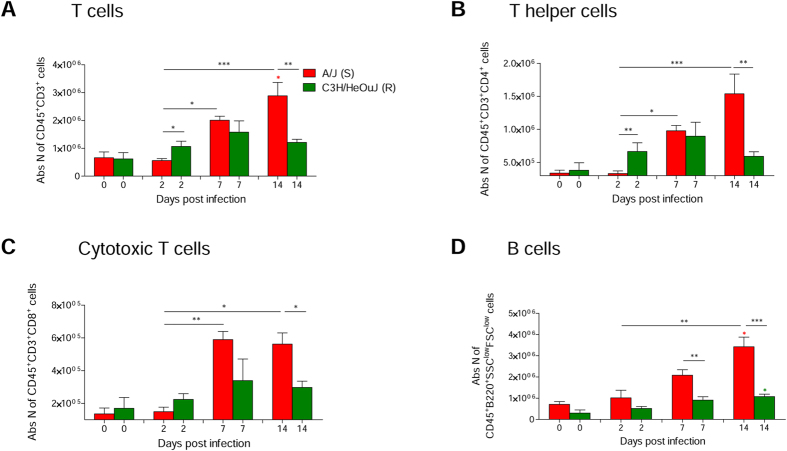
Levels of CD3^+^, CD4^+^, CD8^+^ and B220^+^ cells during time course analysis of chronic *P. aeruginosa* infection in resistant and susceptible mice. Susceptible A/J and resistant C3H/HeOuJ mice were inoculated with 2 × 10^6^ CFU of *P. aeruginosa* mucoid clinical isolate AA43 embedded in agar beads by intratracheal injection and sacrificed at day 2, 7 and 14 post challenge. The absolute numbers of T cells (CD45^+^CD3^+^) (**A**), T helper cells (CD45^+^CD3^+^CD4^+^) (**B**), cytotoxic T cells (CD45^+^CD3^+^CD8^+^) (**C**) and B lymphocytes (B220^+^ SSC^low^ FSC^low^) (**D**) were measured by flow cytometric in lung cell suspension analysis of naïve A/J and C3H/HeOuJ mice and after 2, 7 and 14 days post challenge and represented as bars. Bars represent mean values and the error bars the SEM. The data are pooled from two independent experiments. Statistical significance by Mann-Whitney U test and nonparametric Kruskal-Wallis test followed by post-hoc Dunn test to correct for multiple comparisons is indicated: *p < 0.05, **p < 0.01, ***p < 0.001. Colored stars indicate the statistical significance of the difference between each mouse strain at the specific time point compared with its own naïve counterpart.

T cells orchestrate the activity of neutrophils and alveolar macrophages over the course of *P. aeruginosa* infection^23^. We found that at day 2, CD3^+^ and CD4^+^ T cell infiltrated the infected lungs to higher extents in C3H/HeOuJ resistant mice than in A/J susceptible ones (Fig. 5A,B, Supplementary Fig. 3A,B). By day 7 T cell infiltration increased in C3H/HeOuJ mice and decreased thereafter. In contrast CD3^+^ and CD4^+^ T cell subsets progressively accumulated in A/J mice both at day 7 and day 14, being significantly enriched for when compared to C3H/HeOuJ. Similar trends were observed for CD8^+^ T cells and B cells, accumulating to higher numbers in A/J mice at day 7 and day 14 compared to C3H/HeOuJ ones (Fig. 5C,D, Supplementary Fig. 3C,D). Thus, these data indicate that a superior CD4^+^ T cell responses shortly after infection is recruited in inbred strain with a better survival and resistance to chronic infection; on the other side, a delayed response is associated with susceptibility to *P. aeruginosa* chronic infection.

**Figure 6:**
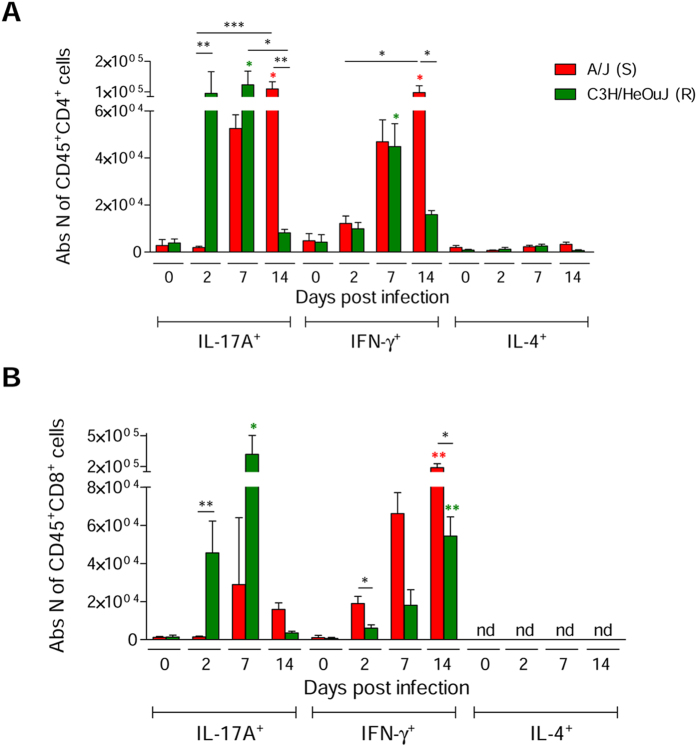
Levels of CD4^+^ and CD8^+^ cells capable of IFNγ, IL17A and IL4 production measured during chronic *P. aeruginosa* infection in resistant and susceptible mice. Susceptible A/J and resistant C3H/HeOuJ mice were inoculated with 2 × 10^6^ CFU of *P. aeruginosa* mucoid clinical isolate AA43 embedded in agar beads by intratracheal injection and sacrificed at day 2, 7 and 14 post challenge. The absolute numbers of IFNγ-, IL17A- and IL4-producing CD45^+^CD4^+^ (**A**) and IFNγ- and IL17A-producing CD45^+^CD8^+^ cells (**B**) were measured in lung cell suspension, after PMA/ionomycin stimulation, by flow cytometric analysis of naïve mice and after 2, 7 and 14 days post challenge and represented as bars. Bars represent mean values and the error bars the SEM. The data are pooled from two independent experiments. Statistical significance by Mann-Whitney U test and nonparametric Kruskal-Wallis test followed by post-hoc Dunn test to correct for multiple comparisons is indicated: *p < 0.05, **p < 0.01, ***p < 0.001. Colored stars indicate the difference between each mouse strain at the specific time point compared with its own naïve counterpart. nd: not detectable.

Of note, within the CD4^+^ cell subset, IL17-producing cells were best represented in C3H/HeOuJ mice early after infection (day 2 and day 7). While IL17^+^ cells decreased by day 14 in C3H/HeOuJ mice, they accumulated to significant numbers in A/J mice, along with IFNγ^+^ cells and not IL4^+^ cells (Fig. 6A, Supplementary Fig. 4A). Of note, also IL17-producing TCRγδ^+^ T cells (Supplementary Fig. 5A) and CD8^+^ cells were selectively enriched for in C3H/HeOuJ and not A/J lungs at day 2 (Fig. 6B, Supplementary Fig. 4B), while no different levels of IL17^+^ neutrophils were observed (Supplementary Fig. 5B).

Thus, the rapid infiltration of the lung by IL17-producing cells appears to promote the accumulation of alveolar macrophages and CD8^+^IL17^+^, favouring bacterial clearance, resistance to chronic infection and improved survival. At difference, suboptimal and delayed CD4^+^IL17^+^ responses suggest to favour neutrophils persistence in infected lungs and chronic inflammation, likely contributing to the establishment of chronic infection.

## Discussions

In this work, we have exploited a mouse model of chronic *P. aeruginosa* infection, and demonstrated that the host genetic background confers different immunoreactivity to the pathogen, contributing to predisposition to chronic infection.

To mimic the progressive bronchopulmonary infection typical of CF and COPD patients and study the impact of the genetic background on the establishment of chronic infections, we challenged five different inbred murine strains with the AA43 *P. aeruginosa* adapted clinical isolate embedded in agar beads^21^,^24^. By evaluating mortality, changes in body weight, the capacity to efficiently clear the pathogen, and the frequency of persistent infection, we defined a susceptibility range and ranked the mice accordingly. We found the A/J, 129S2/SvPasCrl and DBA/2 J strains to be most susceptible to *P. aeruginosa* chronic infection, while the C57BL/6NCrl and C3H/HeOuJ strains to be most resistant. Of note, these results are in line with previous studies^5^,^14^ and with results obtained in acute infection models^9^, the only exception being the C57BL/6NCrl strain, which revealed an intermediate susceptibility to acute infection and instead proved resistant to chronic disease. Thus, in general terms, it appears that host genetic backgrounds predisposing to acute *P. aeruginosa* infections also appear more prone to accommodate long-term chronic bronchopulmonary disease. Translating this observation to CF patients, it is plausible to suggest that the first colonization, successive episodes of re-colonization and later chronic infection are all events influenced by the genetic profile of the host.

To define possible causes for predisposition, we characterized immune-related events possibly linked to diverse *P. aeruginosa* susceptibility and resistance. Early responses to the pathogen (day 2) included lung infiltration by inflammatory phagocytes, neutrophils and macrophages, which occurred to a similar extent in susceptible (A/J) and resistant (C3H/HeOuJ) hosts. Also the *in vitro* phagocytic capacity of lung inflammatory neutrophils and macrophages derived from resistant C3H/HeOuJ and susceptible A/J mice was found to be similar, as also previously reported by Morissette and co-authors in the case of resistant (BALB/c) and susceptible (DBA2) mice^6^. Thus, the early phases of the response to the pathogen by components of the innate immune response appear to be similar between susceptible and resistant host, and could not account for predisposition to development of chronic infection. This observation differs from data generated when studying the AA2 acute infection model, which showed inter-strain differences in the early recruitment of the innate immune response^9^. Differences in the early phases of acute versus chronic infection models might be due to the *P. aeruginosa* clinical isolates adopted in the two studies (the AA2 vs AA43), in the modality of infection (free bacteria vs agar-beads embedded ones) and to the time of analysis (6–18 h *vs* day 2). Further studies will be needed to better define the molecular events subtending innate cell responsiveness in AA2 and AA43 models.

Nevertheless, resistant C3H/HeOuJ mice showed a prevalence of alveolar macrophages in infected lungs at day 2 and day 7. This might favor resolution of inflammation via phagocytosis of dying neutrophils and initiate mechanisms of repair^25^, which were evident in lung histopathological analyses. Accordingly, neutrophils numbers were lower by day 7 and 14 in resistant mice when compared to susceptible A/J, which revealed sustained neutrophil lung infiltration over the course of chronic infection.

When analyzing the contribution of adaptive immune responses, *P. aeruginosa*-resistant and susceptible hosts appeared also clearly different. FACS-assisted immunophenotyping revealed superior lung infiltration by T cells in resistant C3H/HeOuJ mice compared to A/J susceptible ones by day 2 (including CD4^+^, CD8^+^ and TCRγδ^+^ T cell subsets), which were highly enriched for IL17-producing cells. Given the fast kinetic of infiltration, we believe that CD4^+^IL17^+^ cells, that predominate the early phases of *P. aeruginosa* infection, belong to the “natural” Th17 (nTh17) cell subset, described in the gut, liver, oral mucosa and lungs predominate the early phases of *P. aeruginosa* infection. These cells, differently from conventional Th17 (cTh17) cells, which require antigen-driven priming, can be mobilized in hours or days^26^. Our data are in line with other studies, reporting IL17 producing Th17 cells and TCRγδ^+^ T cells as critical in the fast host immune defense against *P. aeruginosa*^27^. Whether other cellular sources (e.g. pulmonary group 3 innate lymphoid cells and natural killer cells), previously described to be critical in other models of *P. aeruginosa* infection^28^, also contribute to the early phases of chronic infection, remains to be determined.

Previous studies had defined Th1-like cells as critical against *P. aeruginosa* infection. Indeed, Th1-prone C3H/HeN mice were reported to have a better disease outcome when compared to the Th2-prone BALB/c mice^14^,^15^. In our work, while IL4-producing CD4^+^ T cells were negligible in both resistant and susceptible mice, we detected equal levels of IFNγ-producing CD4^+^ T cells in response to the pathogen. Instead, we found the early IL17-dominated response best observed in resistant mice is better associated with the efficient clearance of *P. aeruginosa* and the progressive resolution of inflammation, along with the protection against chronic infection, and an overall milder clinical outcome. Compared to resistant C3H/HeOuJ mice, susceptible A/J mice showed a delayed infiltration of the lung by CD4^+^IL17^+^ cells and a more prolonged CD4^+^IFNγ^+^ cell response. Results obtained in susceptible mice are highly reminiscent of those we recently reported in the C57BL/6NCrl background^17^, and support a model by which IL17 might provide a protective role in early phases of infection, and instead might be detrimental afterwards and sustain immunopathological manifestations when chronically produced in the context of inflammation. In a recent study, we found that IL-17 is active at different phases of chronic airways infection and indeed plays a double-edged activity^17^,^28^. On one side we found IL-17 to contribute to the control of *P. aeruginosa* burden, while on the other, IL-17 appeared to worsen pulmonary neutrophilia and tissue damage. We believe that the current study supports a previous report and a model whereby IL-17 produced by neutrophils and CD4^+^ T cells plays a critical role in the early phase of infection by contributing to pathogen clearance. In the case of delayed recruitment of such cells, bacterial persistence causes abnormal accumulation of these cells, later on responsible for chronic inflammation and tissue damage.

Thus, overall our results indicate that the host genetic background defines distinct immune-reactivity to *P. aeruginosa* infection, and consequently a diverse susceptibility to chronic infections. Our results suggest a key role for IL17-producing cells in the early phases of infections. Mice with pre-existing IL17-producing cells are most likely to eradicate the pathogen at the time of first encounter, and to resolve pathogen-induced inflammation, enabling limited pathological manifestations. Thus, strategies capable to increase the frequency of IL17^+^ cells might be envisaged to protect patients against *P. aeruginosa*-induced chronic infection, especially in CF. Conversely, prolonged IL17 expression correlated with chronicity of infection and likely exacerbated immunopathology. This knowledge can be further explored in patients to assess the risk of *P. aeruginosa* chronic infection based on their genetic profile or treatments inhibiting prolonged IL17 expression.

## Materials and Methods

### Bacterial strains

The AA43 *P. aeruginosa* clinical strain was originally isolated at a late stage of chronic infection from a CF patient^22^,^29^, cultured in trypticase soy broth (TSB) and plated on trypticase soy agar (TSA).

### Mouse model of *P. aeruginosa* chronic infection

Ten-twelve weeks old inbred mice were used: A/J, C3H/HeOuJ and DBA/2 J were purchased from Jackson Laboratories, C57BL/6NCrl and 129S2/SvPasCRL were obtained from Charles River Laboratories. Mice were infected with 2 × 10^6^ CFU of *P. aeruginosa* embedded in agar beads, as previously described^22^,^24^. Animals were sacrificed after 2, 7 and 14 days by CO_2_ administration and lungs were recovered.

Animal studies were conducted according to protocols approved by San Raffaele Scientific Institute (Milan, Italy) Institutional Animal Care and Use Committee (IACUC) (Permit number: 502).

### Lung single-cell suspension and flow cytometric analysis

Lung single-cell suspensions were obtained by mashing the lungs through a 70-μM cell strainer in RPMI + 5% FBS and serially diluted and plated on TSA for CFU counts. All the procedures were previously described^17^. Briefly, lung cell suspensions (1–3 × 10^6^ cells) were incubated with blocking buffer (5% rat serum and 95% culture supernatant of 24G2 anti-FcR mAb-producing hybridoma cells) for 10 min at 4 °C. Then, cells were stained for 20 min at 4 °C in the darkness with different combinations of antibodies (BD Biosciences; listed in Supplementary Table 1). For intracellular cytokine staining, 1–3 × 10^6^ cells were stimulated with PMA (50 ng/ml) and Ionomycin (1 μg/ml) for 4 h at 37 °C (the last 2 h with Brefeldin A (5 μg/ml)). Cells were surface stained, fixed, permeabilized and then stained for intracellular cytokines for 30 min at RT in darkness. Cells were washed in permeabilization buffer and collected using a FACSCANTO flow cytometry (BD Biosciences) and then analyzed using FlowJo software.

### Histologic and immunofluorescence analysis

Lungs were embedded in paraffin and 2-mm sections were stained by Haematoxylin-Eosin for histological analysis. De-paraffinized lung sections were stained with rabbit antiserum specific for *P. aeruginosa* and Texas Red-labelled goat anti-rabbit IgG as described previously for bacterial localization^22^. The slides were examined using an Axioplan fluorescence microscope (Zeiss).

### Cytokine analysis

Part of the harvested lung single-cell suspensions, obtained by mechanical dissociation, were centrifuged at 14000 rpm for 30 min at 4 °C and the supernatants (SN) were stored at −80 °C for quantification of total protein content with Bradford’s assay (Bio-RAD). Protein content was quantified at the final concentration of 500 μg/ml. A panel of murine chemokines and cytokines were measured using Bio-Plex pro^TM^ Mouse Cytokine Standard 23-Plex, Group I and Bio-Plex pro^TM^ Mouse Cytokine Custom TH17 Mouse Group III.

### Phagocytosis and intracellular killing assay

Bone marrow cells were isolated from naïve mice and neutrophils were negatively selected with STEMCELL mouse enrichment Kit (catalog #19762)^30^. Peritoneal cells were harvested 3 days after i.p. injection of 1 ml thioglycolate broth (BD) by two lavages with 5 ml PBS. The purity of cells determined by Diff-Quick staining was 85–90%.

For neutrophils infection, *P. aeruginosa* cells where opsonized with 10% mouse serum for 30 min at room temperature in agitation. Both neutrophils and macrophages were infected with *P. aeruginosa* AA43 at mid exponential phase, with a MOI of 100:1 (bacteria:neutrophils). After 30 min of incubation at 37 °C cells were treated with Polymvxins B (100 μg/ml) (Sigma), washed, lysed with H_2_0 and plated on TSA. To evaluate intracellular killing capacity, treatment with Polymvxins B was extended for 30 min, 60 min and 90 min for macrophages.

### Statistical analysis

Data analysis was performed using a nonparametric two-taled Mann-Whitney U test for single comparisons, such as when comparing data between two murine strains at a specific time-point (e.g. CFU levels). To compare data for a specific murine strain across multiple time-points a nonparametric Kruskal-Wallis test was used followed by post-hoc Dunn test to correct for multiple comparisons. Rates of mortality and infection were compared using Fisher’s test followed by Bonferroni correction for multiple comparisons. Mantel–Cox test was used to compare survival between pairs. The changes in body weight were compared using Two-way ANOVA with Bonferroni’s post-hoc test. *P* < 0.05 was considered significant.

## Additional Information

**How to cite this article**: Spagnuolo, L. *et al.* The host genetic background defines diverse immune-reactivity and susceptibility to chronic **Pseudomonas aeruginosa** respiratory infection. *Sci. Rep.* **6**, 36924; doi: 10.1038/srep36924 (2016).

**Publisher’s note:** Springer Nature remains neutral with regard to jurisdictional claims in published maps and institutional affiliations.

## Supplementary Material

Supplementary Information

## Figures and Tables

**Table 1:** Cytokines and chemokines levels in lungs of susceptible A/J and resistant C3H/HeOuJ mice during time course of *P. aeruginosa* chronic lung infection.

Cytokines	naïve		Day2		Day7		Day14		A/J vs C3H/HeOuJ			
	A/J	C3H/HeOuJ	A/J	C3H/HeOuJ	A/J	C3H/HeOuJ	A/J	C3H/HeOuJ	naive	Day2	Day7	Day14
IL1α	0,54	1,55	**205,70**	150,30	**201,20**	50,38	18,88	12,95	ns	ns	*	ns
IL1β	10,73	3,80	214,60	**116,70**	**322,70**	**90,37**	25,76	20,62	ns	*	**	ns
IL6	0,62	0,62	**11,94**	0,62	2,99	0,62	0,62	0,62	ns	*	ns	ns
MCP1	11,10	1,24	**191,80**	49,27	6,87	9,12	1,24	1,24	ns	*	ns	ns
MIP1α	0,10	0,10	**165,00**	**96,38**	**72,71**	27,50	4,30	1,69	ns	*	*	ns
MIP1β	9,10	9,44	**66,05**	**32,07**	47,07	22,59	16,97	15,82	ns	*	*	ns
TNFα	3,16	3,16	**12,05**	3,16	3,25	3,16	3,16	3,16	ns	ns	ns	ns
KC	6,61	7,13	**181,30**	**102,00**	**73,10**	33,49	13,84	7,91	ns	*	ns	ns
G-CSF	3,84	1,93	**386,80**	126,70	31,01	18,63	5,06	2,71	ns	*	ns	ns
IFNγ	nd	nd	nd	nd	nd	nd	nd	nd	—	—	—	—
IL2	nd	nd	nd	nd	nd	nd	nd	nd	—	—	—	—
IL12p70	1,74	1,74	**53,63**	**17,61**	14,32	1,74	1,74	1,74	ns	ns	*	ns
IL17A	4,44	4,54	8,63	**8,10**	**20,53**	7,13	6,44	7,50	ns	ns	**	ns
IL12p40	1,62	1,77	57,61	20,32	**109,00**	**36,01**	25,95	9,69	ns	*	**	ns
IL23p19	0,04	0,04	0,09	0,08	0,09	0,05	**0,10**	0,08	—	—	*	—
IL21	0,06	0,05	0,09	0,08	**0,10**	0,07	**0,10**	0,09	—	—	*	—
IL22	0,00	0,00	0,01	0,01	0,01	0,01	0,01	0,01	—	—	—	—
IL10	0,60	0,31	**11,93**	**5,34**	3,90	2,01	0,92	1,70	ns	*	ns	ns

Data are expressed as mean of pg/500 μg lung. Statistical analysis for comparison of A/J vs C3H/HeOuJ by the non-parametric Mann-Whitney U test and by nonparametric Kruskal-Wallis test is indicated followed by post-hoc Dunn test to correct for multiple comparisons for a specific murine strain across multiple time-points (*p < 0.05, **p < 0,01) is reported. In bold significance of each value compared with its naïve counterpart. nd: not detectable; ns: not significant.
