# Supplementary material for: The host genetic background defines diverse immune-reactivity and susceptibility to chronic Pseudomonas aeruginosa respiratory infection
Source: Sci Rep. 2016 Nov 16;6:36924. doi: 10.1038/srep36924 (PMC5111113; doi:10.1038/srep36924)
Supplement: Supplementary Information [file srep36924-s1.doc]

**The host genetic background defines diverse immune-reactivity and susceptibility to chronic *Pseudomonas aeruginosa* respiratory infection**

Spagnuolo Lorenza^1^**^#^**, De Simone Maura^1^**^#^**, Lorè Nicola Ivan^1^**^#^**, De Fino Ida^1^, Basso Veronica^2^, Mondino Anna^2^, Cigana Cristina^1&^, Bragonzi Alessandra^1&^

Supplementary Information

**Supplementary Table S1.** Antibodies used for FACS analysis and intracellular staining are indicated.

| **Antibodies panel** |
| --- |
| CD45- PacBlue (clone30-F11) |
| Gr-1- FITC (clone RB6-8C5) |
| CD11b- APC (clone M1/70) |
| CD11c- PeCy7 (clone N418) |
| CD3- APC (clone 145-2C11) |
| B220- PerCP (clone RA3-6B2) |
| CD4- PE (clone RM4-5) |
| CD4 – PerCP (clone RM4-5) |
| CD8- PeCy7 (clone 53-6.7) |
| CD44- FITC (clone IM7) |
| TCRγδ -APC (cloneGL3) |
| IL17 -PE (clone TC11-18H10.1) |
| IFNγ- FITC (clone XMG1.2) |
| IL4- APC (clone 11B11) |

**Supplementary Table S2.** Statistical comparison of change in body weight among inbred mice infected with *P. aeruginosa*.

|  |  | **A/J** | **C3H/HeOuJ** | **C57BL/6NCrl** | **DBA2/J** |
| --- | --- | --- | --- | --- | --- |
| **Day1** | **A/J** |  |  |  |  |
|  | **C3H/HeOuJ** | ns |  |  |  |
|  | **C57BL/6NCrl** | ns | ns |  |  |
|  | **DBA2/J** | ns | ns | ns |  |
|  | **129S2/SvPasCrl** | ns | ns | ns | ns |
| **Day2** | **A/J** |  |  |  |  |
|  | **C3H/HeOuJ** | ** |  |  |  |
|  | **C57BL/6NCrl** | ns | ns |  |  |
|  | **DBA2/J** | ns | * | ns |  |
|  | **129S2/SvPasCrl** | ns | ns | ns | ns |
| **Day3** | **A/J** |  |  |  |  |
|  | **C3H/HeOuJ** | **** |  |  |  |
|  | **C57BL/6NCrl** | **** | ns |  |  |
|  | **DBA2/J** | ns | ** | ** |  |
|  | **129S2/SvPasCrl** | ns | **** | **** | ns |
| **Day 4** | **A/J** |  |  |  |  |
|  | **C3H/HeOuJ** | *** |  |  |  |
|  | **C57BL/6NCrl** | **** | ns |  |  |
|  | **DBA2/J** | ns | ns | *** |  |
|  | **129S2/SvPasCrl** | ns | *** | **** | ns |
| **Day 5** | **A/J** |  |  |  |  |
|  | **C3H/HeOuJ** | **** |  |  |  |
|  | **C57BL/6NCrl** | **** | ns |  |  |
|  | **DBA2/J** | ns | ns | **** |  |
|  | **129S2/SvPasCrl** | ns | *** | **** | ns |
| **Day6** | **A/J** |  |  |  |  |
|  | **C3H/HeOuJ** | **** |  |  |  |
|  | **C57BL/6NCrl** | **** | ns |  |  |
|  | **DBA2/J** | ns | * | **** |  |
|  | **129S2/SvPasCrl** | ns | * | **** | ns |
| **Day7** | **A/J** |  |  |  |  |
|  | **C3H/HeOuJ** | ** |  |  |  |
|  | **C57BL/6NCrl** | **** | ns |  |  |
|  | **DBA2/J** | ns | ns | *** |  |
|  | **129S2/SvPasCrl** | ns | ns | **** | ns |

|  |  | **A/J** | **C3H/HeOuJ** | **C57BL/6NCrl** | **DBA2/J** |
| --- | --- | --- | --- | --- | --- |
| **Day8** | **A/J** |  |  |  |  |
|  | **C3H/HeOuJ** | **** |  |  |  |
|  | **C57BL/6NCrl** | **** | ns |  |  |
|  | **DBA2/J** | ns | ns | *** |  |
|  | **129S2/SvPasCrl** | ns | ns | **** | ns |
| **Day9** | **A/J** |  |  |  |  |
|  | **C3H/HeOuJ** | **** |  |  |  |
|  | **C57BL/6NCrl** | **** | ns |  |  |
|  | **DBA2/J** | ns | ns | ** |  |
|  | **129S2/SvPasCrl** | ns | ns | **** | ns |
| **Day10** | **A/J** |  |  |  |  |
|  | **C3H/HeOuJ** | *** |  |  |  |
|  | **C57BL/6NCrl** | **** | * |  |  |
|  | **DBA2/J** | ns | ns | ** |  |
|  | **129S2/SvPasCrl** | ns | ns | **** | ns |
| **Day11** | **A/J** |  |  |  |  |
|  | **C3H/HeOuJ** | **** |  |  |  |
|  | **C57BL/6NCrl** | **** | ns |  |  |
|  | **DBA2/J** | ns | ns | ns |  |
|  | **129S2/SvPasCrl** | ns | ns | **** | ns |
| **Day12** | **A/J** |  |  |  |  |
|  | **C3H/HeOuJ** | **** |  |  |  |
|  | **C57BL/6NCrl** | **** | ns |  |  |
|  | **DBA2/J** | ns | ns | *** |  |
|  | **129S2/SvPasCrl** | ns | * | **** | ns |
| **Day13** | **A/J** |  |  |  |  |
|  | **C3H/HeOuJ** | **** |  |  |  |
|  | **C57BL/6NCrl** | **** | ns |  |  |
|  | **DBA2/J** | ns | ns | ** |  |
|  | **129S2/SvPasCrl** | ns | ** | **** | ns |
| **Day14** | **A/J** |  |  |  |  |
|  | **C3H/HeOuJ** | **** |  |  |  |
|  | **C57BL/6NCrl** | **** | ns |  |  |
|  | **DBA2/J** | ns | ns | ** |  |
|  | **129S2/SvPasCrl** | ns | ** | **** | ns |

Two way ANOVA test was used for pair wise comparisons between inbred mouse strains at different days (*p<0.05, **p<0.01, ***p<0.001, ****p<0.0001, ns: not significant).

**Supplementary Table S3.** Survival, chronic infection and bacterial load in the lungs in inbred mice infected with *P. aeruginosa*.

| **Mouse strain** | **No. of mice^a^** | **Mortality % (No. of dead/total mice)** | **Chronic infection % (No. of infected/surviving mice)^b^** | **CFU/lung of surviving mice ^c^** |
| --- | --- | --- | --- | --- |
| **C57BL6NCrl** | 11 | 1 (1/11) | 40 (4/10) | 7,33 x 10^3^ |
| **C3H/HeOuJ** | 26 | 4 (1/26) | 17 (4/23) | 8,50 x 10^3^ |
| **A/J** | 24 | 26 (6/23) | 56 (9/16) | 4,18 x 10^4^ |
| **DBA/2J** | 10 | 30 (3/10) | 71 (5/7) | 4,48 x 10^4^ |
| **129S2/SvPasCrl** | 10 | 30 (3/10) | 71 (5/7) | 3,89 x 10^4^ |

(a) Pooled mice, analyzed in two to three independent experiments, (b) infected mice, surviving 14 days after challenge, (c) data represent median values of mice, chronically infected at 14 days after challenge.

**SUPPLEMENTARY FIGURES AND LEGENDS**


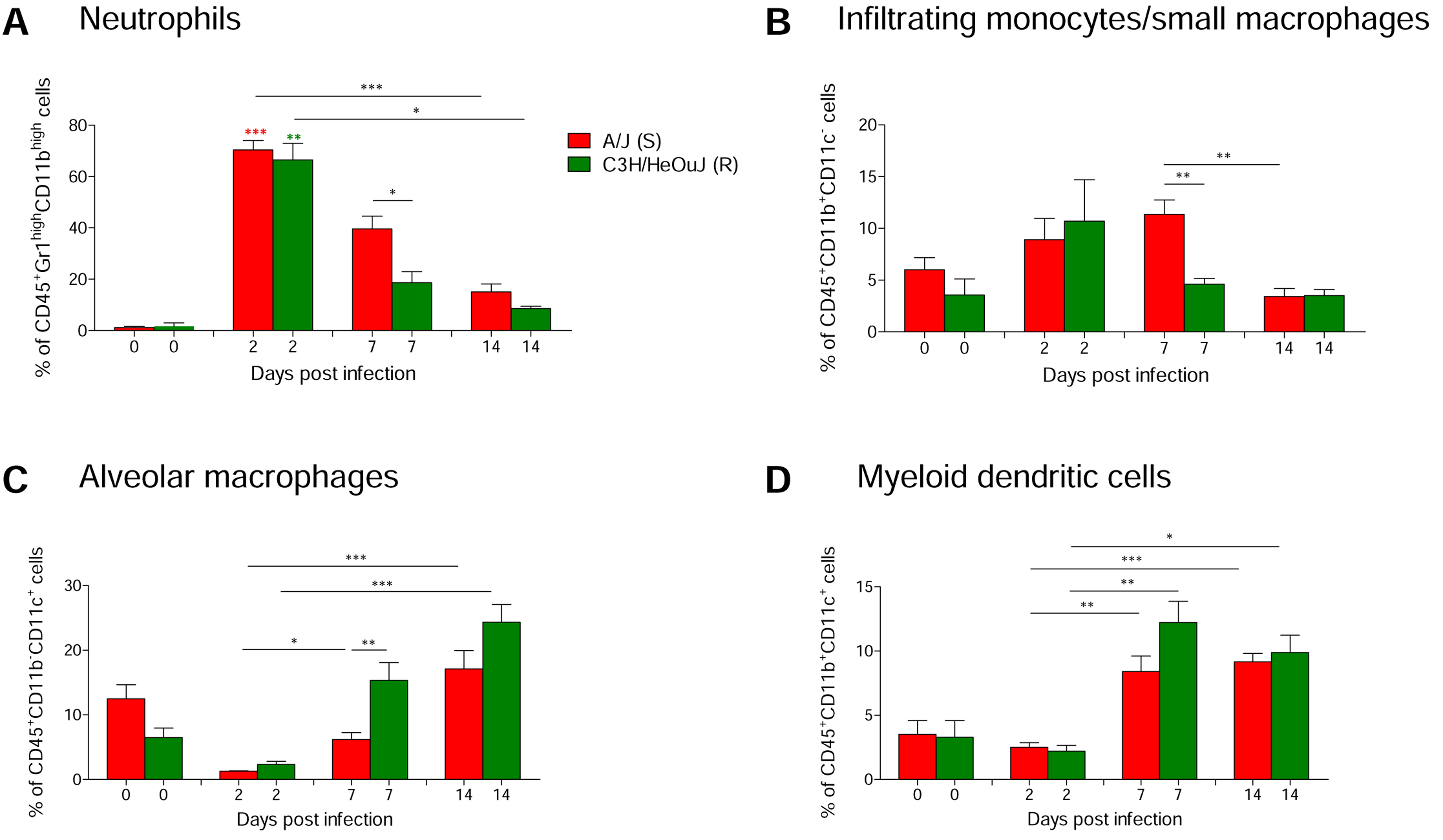


**Figure S1: Frequencies of neutrophils, infiltrating monocytes/small macrophages, alveolar macrophages and myeloid dendritic cells during chronic *P. aeruginosa* infection in resistant and susceptible mice**. Susceptible A/J and resistant C3H/HeOuJ mice were inoculated with 2x10^6^ CFU of *P. aeruginosa* mucoid clinical isolate AA43 embedded in agar beads by intratracheal injection and sacrificed at day 2, 7 and 14 post challenge. The frequencies of neutrophils (CD45^+^Gr1^high^CD11b^high^) (**A**), infiltrating monocytes/small macrophages (CD45^+^CD11b^+^CD11c^-^) (**B**), alveolar macrophages (CD45^+^CD11b^-^ CD11c^+^) (**C**) and myeloid dendritic cells (mDCs) (CD45^+^CD11b^+^CD11c^+^) (**D**) were measured by flow cytometric analysis in lung cell suspension of naïve mice and after 2, 7 and 14 days post challenge and represented as bars. Bars represent mean values and the error bars the standard error of the mean (SEM). The data are pooled from two independent experiments. Statistical significance by Mann-Whitney U test and nonparametric Kruskal-Wallis test followed by post-hoc Dunn test to correct for multiple comparisons is indicated: *p<0.05, **p<0.01, ***p<0.001. Colored stars indicate the statistical significance of the difference between each mouse strain at the specific time point compared with its own naïve counterpart.

**
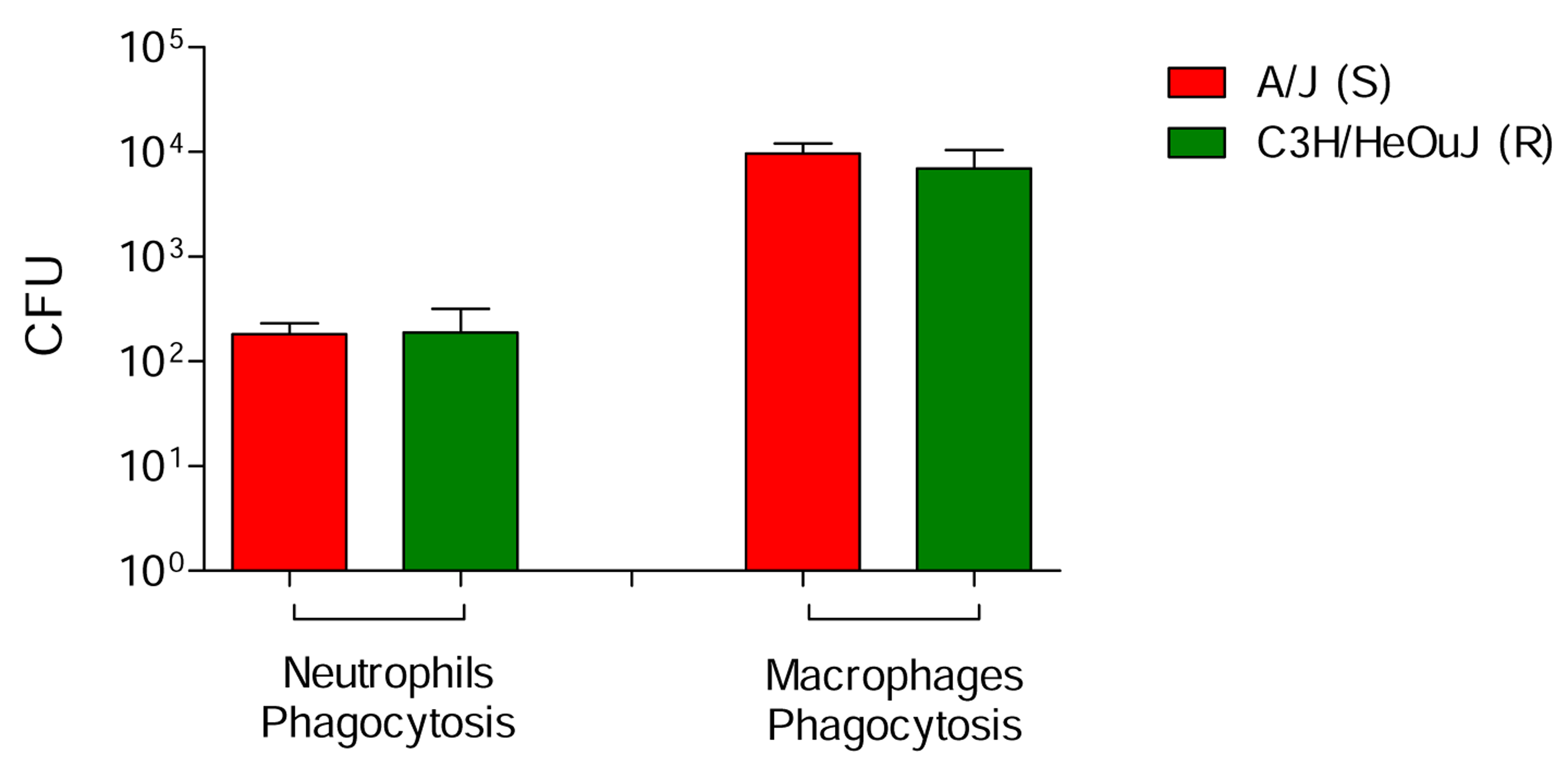
**

**Figure S2: *In vitro* ability of neutrophils and macrophages to phagocyte and kill *P. aeruginosa* in resistant and susceptible mice.** Bone marrow neutrophils and peritoneal macrophages were isolated from naïve C3H/HeOuJ and A/J mice and *in vitro* infected with the *P. aeruginosa* strain AA43, previously opsonized with the serum of the specific inbred strain. Bars represent mean values and the error bars the SEM. The data are pooled from three independent experiments.


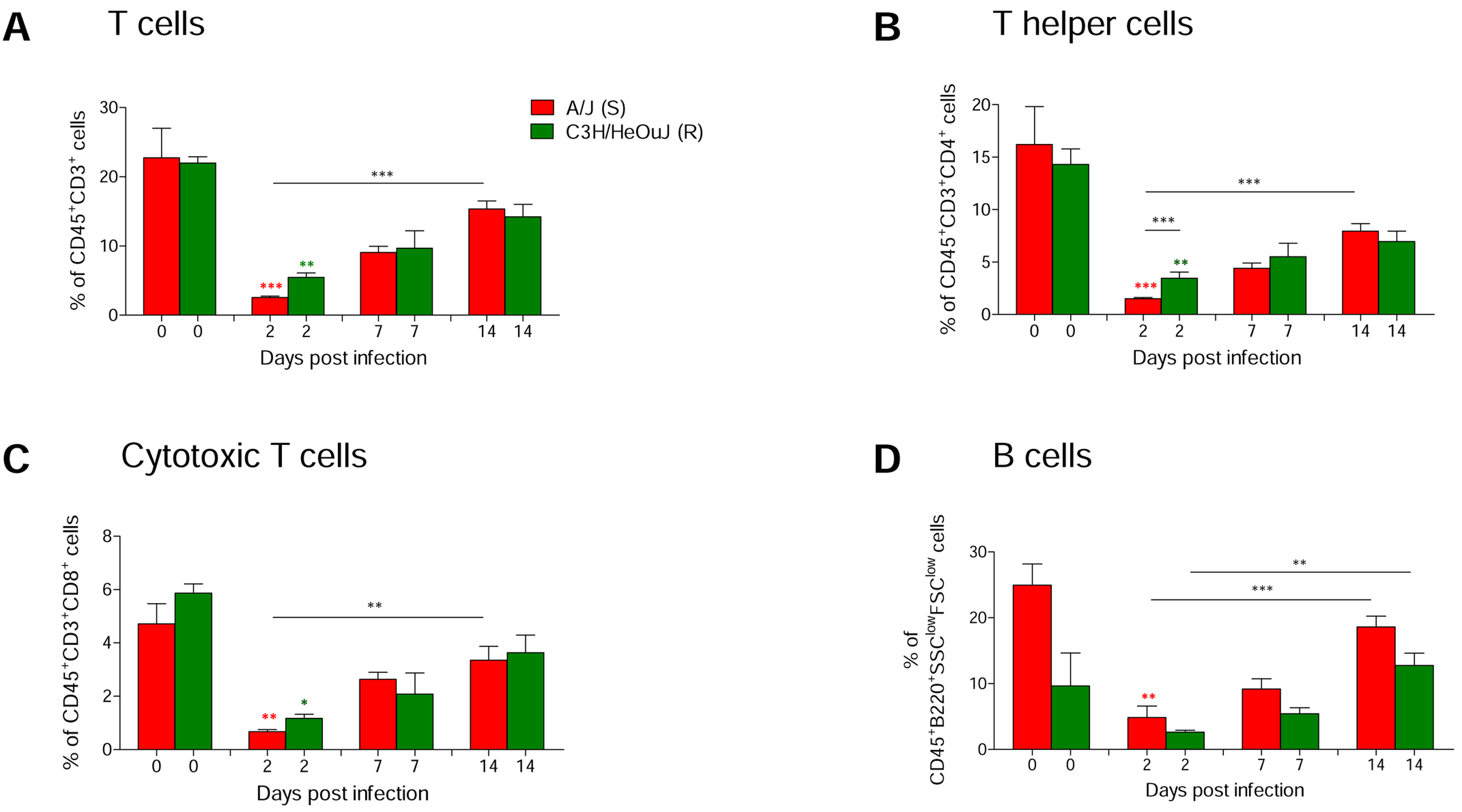


**Figure S3: Frequencies of CD3^+^, CD4^+^, CD8^+^ and B220^+^ cells during time course analysis of chronic *P. aeruginosa* infection in resistant and susceptible mice**. Susceptible A/J and resistant C3H/HeOuJ mice were inoculated with 2x10^6^ CFU of *P. aeruginosa* mucoid clinical isolate AA43 embedded in agar beads by intratracheal injection and sacrificed at day 2, 7 and 14 post challenge. The frequencies of T cells (CD45^+^CD3^+^) (**A**), T helper cells (CD45^+^CD3^+^CD4^+^) (**B**), cytotoxic T cells (CD45^+^CD3^+^CD8^+^) (**C**) and B lymphocytes (B220^+^ SSC^low^ FSC^low^) (**D**) were measured by flow cytometric in lung cell suspension analysis of naïve A/J and C3H/HeOuJ mice and after 2, 7 and 14 days post challenge and represented as bars. Bars represent mean values and the error bars the SEM. The data are pooled from two independent experiments. Statistical significance by Mann-Whitney U test and nonparametric Kruskal-Wallis test followed by post-hoc Dunn test to correct for multiple comparisons is indicated: *p<0.05, **p<0.01, ***p<0.001. Colored stars indicate the statistical significance of the difference between each mouse strain at the specific time point compared with its own naïve counterpart.


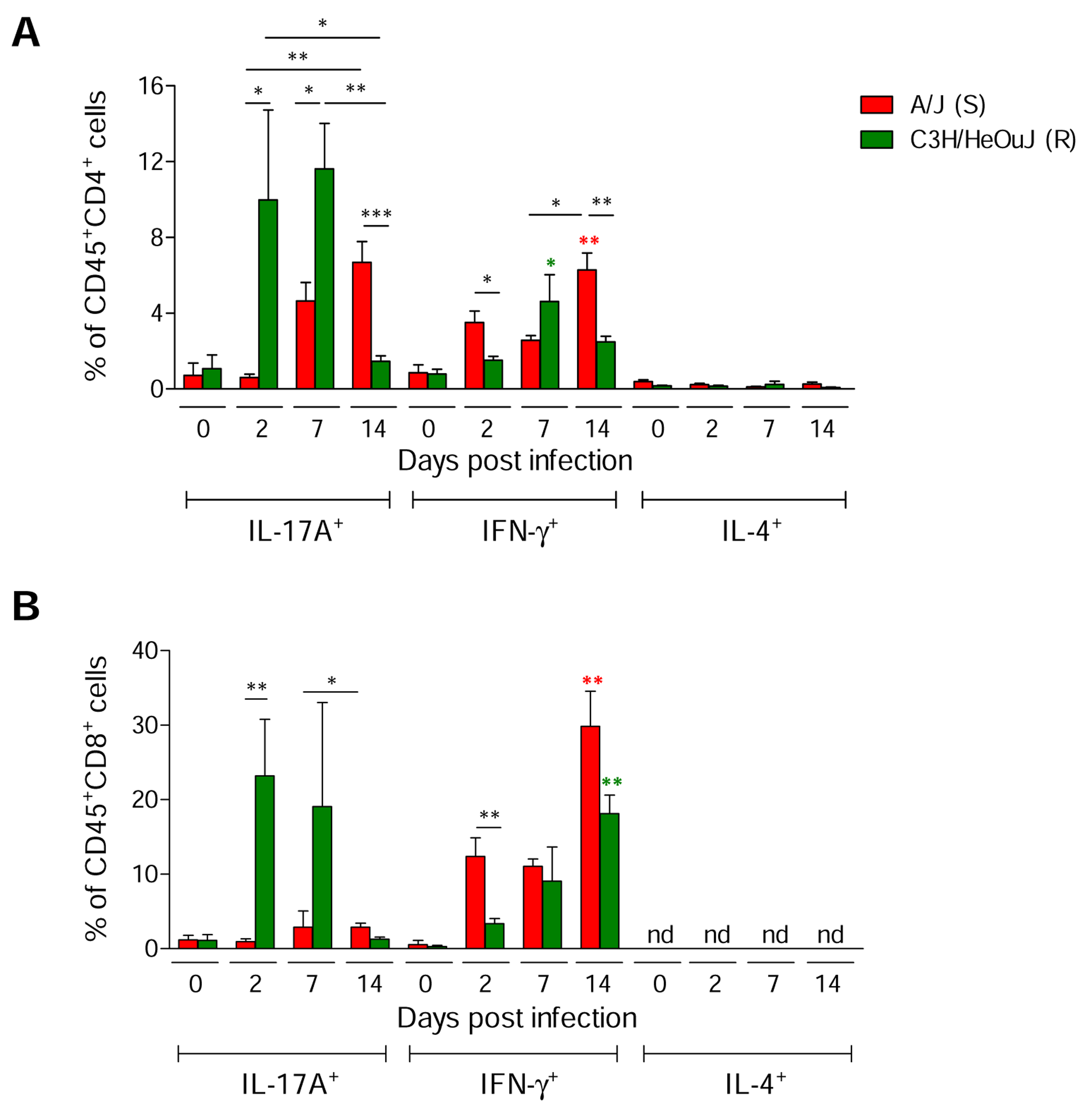


**Figure S4: Frequencies of CD4^+^ and CD8^+^ T cells capable of IFNγ, I-17A and IL4 production measured during chronic *P. aeruginosa* infection in resistant and susceptible mice.** Susceptible A/J and resistant C3H/HeOuJ mice were inoculated with 2x10^6^ CFU of *P. aeruginosa* mucoid clinical isolate AA43 embedded in agar beads by intratracheal injection and sacrificed at day 2, 7 and 14 post challenge. The frequencies of IFNγ-, IL17A- and IL4-producing CD45^+^CD4^+^ cells (**A**) and IFNγ- and IL17A-producing CD45^+^CD8^+^ cells (**B**) were measured in lung cell suspension, after PMA/ionomycin stimulation, by flow cytometric analysis of naïve mice and after 2, 7 and 14 days post challenge and represented as bars. Bars represent mean values and the error bars the SEM. The data are pooled from two independent experiments. Statistical significance by Mann-Whitney U test and nonparametric Kruskal-Wallis test followed by post-hoc Dunn test to correct for multiple comparisons is indicated: *p<0.05, **p<0.01, ***p<0.001. Coloured stars indicate the difference between each mouse strain at the specific time point compared with its own naïve counterpart. nd: not detectable.


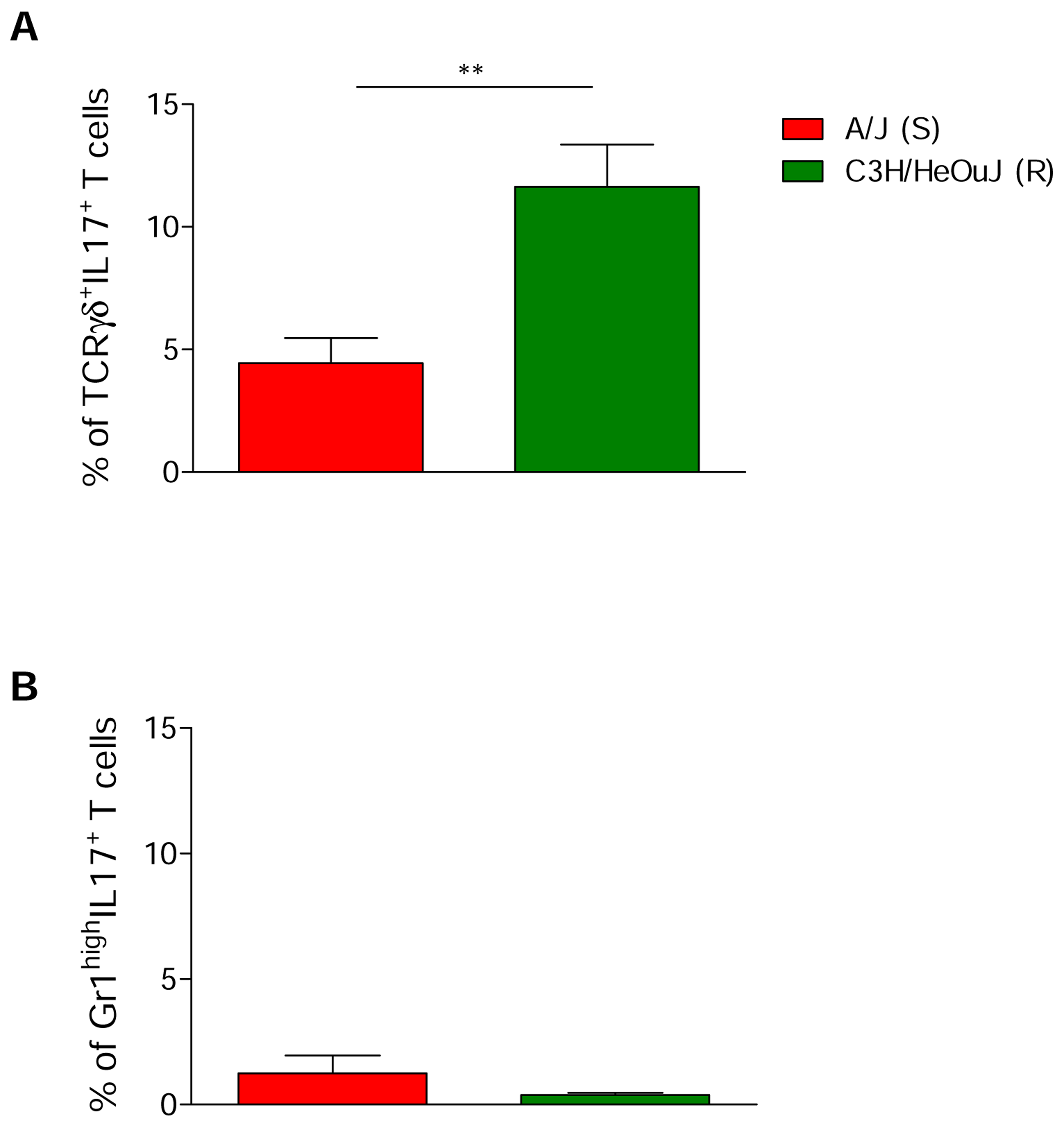


**Figure S5. Frequencies of IL17-producing TCRγδ^+^ T cells and neutrophils after two days of *P. aeruginosa* infection.** Susceptible A/J and resistant C3H/HeOuJ mice were inoculated with 2x10^6^ CFU of *P. aeruginosa* mucoid clinical isolate AA43 embedded in agar beads by intratracheal injection and sacrificed at day 2 post challenge. The frequencies of IL17A-producing TCRγδ^+^ T cells (CD45^+^FSC^low^SSC^low^TCRγδ^+^) (**A**) and neutrophils (CD45^+^Gr1^high^) (**B**) were measured by flow cytometric analysis in lung cell suspension after PMA/ionomycin stimulation. Bars represent mean values and the error bars the SEM. The data are pooled from two independent experiments. Statistical significance by Mann-Whitney U test is indicated: **p<0.01.
